# Don’t turn your back on the symptoms of psychosis: the results of a proof-of-principle, quasi-experimental intervention to reduce duration of untreated psychosis

**DOI:** 10.1186/s12888-016-0816-7

**Published:** 2016-05-04

**Authors:** Charlotte Connor, Max Birchwood, Nick Freemantle, Colin Palmer, Sunita Channa, Clare Barker, Paul Patterson, Swaran Singh

**Affiliations:** University of Warwick, Warwick, UK; University College London, London, UK; Birmingham & Solihull Mental Health NHS Trust, Centre for Mental Health The Barberry Centre, 25 Vincent Drive, Birmingham, B15 2FG UK; Birmingham Children’s Hospital, Birmingham, UK

## Abstract

**Background:**

No evidence based approach to reduce duration of untreated psychosis (DUP) has been effective in the UK. Existing interventions have many components and have been difficult to replicate. The majority of DUP in Birmingham, UK is accounted for by delays within mental health services (MHS) followed by help-seeking delay and, we hypothesise, these require explicit targeting. This study examined the feasibility and impact of an intervention to reduce DUP, targeting help-seeking and MHSs delays.

**Methods:**

A dual-component intervention, comprising a direct care pathway, for 16-25 year olds, and a community psychosis awareness campaign, using our youth-friendly website as the central hub, was implemented, targeting the primary sources of care pathway delays experienced by those with long DUP. Evaluation, using a quasi-experimental, design compared DUP of cases in two areas of the city receiving early detection vs detection as usual, controlling for baseline DUP in each area.

**Results:**

DUP in the intervention area was reduced from a median 71 days (mean 285) to 39 days (mean 104) following the intervention, with no change in the control area. Relative risk for the reduction in DUP was 0.74 (95 % CI 0.35 to 0.89; *p* = .004). Delays in MHSs and help-seeking were also reduced.

**Conclusions:**

Our targeted approach appears to be successful in reducing DUP and could provide a generalizable methodology applicable in a variety of healthcare contexts with differing sources of delay. More research is needed, however, to establish whether our approach is truly effective.

**Trial registration:**

ISRCTN45058713 - 30 December 2012.

**Electronic supplementary material:**

The online version of this article (doi:10.1186/s12888-016-0816-7) contains supplementary material, which is available to authorized users.

## Background

The delay between the onset of a first episode of psychosis and receipt of treatment (duration of untreated psychosis: DUP) has been well documented; mean DUP ranges between 364 to 721 days in different studies [1]. This is of concern because longer DUP has been consistently shown to predict poorer outcome, with some studies suggesting that the first 6-months of treatment delay is a critical period beyond which treatment response and recovery is impaired [2–8]. However, no effective strategy to reduce DUP has been implemented in the UK, even though DUP reduction is a UK Department of Health target, including the introduction of a waiting time standard of no more than two weeks following referral to MHSs [9].

Multi-component interventions have shown the greatest promise in reducing DUP, for example the Norwegian TIPS study [10]; however, similar interventions have failed to replicate these findings in other healthcare contexts [11]. Similar initiatives in Australia [12] aimed at improving help-seeking and recognition of psychosis by targeting schools, and in Canada [13], using active case detection and promotional material targeted at the broader community, for example, have failed to demonstrate any impact on DUP. It has been argued that this is because DUP is not a unitary variable easily targeted by universal means comprising of several component delays (help-seeking delays, referral delays and delays within MHSs) that may each require different strategies [14]. The breakthrough TIPS study [10] used such a *delay-specific* approach, aimed at reducing delays within MHSs and improving awareness of, and help-seeking for, psychosis using a comprehensive education and early detection system. This strategy resulted in TIPS successfully reducing DUP in the intervention area, compared with areas providing detection as usual; DUP was significantly shorter and associated with improved clinical status of clients at the first episode and there was reduced early suicide risk and fewer negative symptoms at 12 months, with positive effects on clinical and functional status maintained at 5 year follow-up [15].

In light of these results, we closely examined the care pathways of young people accepted into Early Intervention Service (EIS) who had long DUP (>6 months), in order to fully understand the sources of delay. EIS teams are specialized services for young people between the ages of 14–35 who have had a first-episode of psychosis which has not previously been treated. EIS are accessed via referral from primary care, for example, a General Practitioner (GP), or from within the same secondary mental health care such as a Community mental health team (CMHT) or Child & Adolescent MHS (CAMHS). These secondary mental health care teams offer multidisciplinary, multi-agency assessment, treatment and care for those with a wide range of mental health problems.

Our previous research identified two principal sources of delay for those young people with long DUP: median *help-seeking delay*s of 66 days (mean 254.6 days) and *delay within MHSs* of 141 days (mean 292.6 days*)* [16]. While their help-seeking delays were often idiosyncratic and difficult to unravel, we discovered direct evidence that delays within MHSs were strongly linked to the nature of the first contact with the secondary mental health services. Those in contact with CMHTs or CAMHs frequently disengaged and subsequently discharged from these services prior to referral to EIS, thus lengthening their DUP. We argued that this arose due to insensitivity of these services to youth and inability to outreach those who disengage.

Informed by these findings, we launched a proof of concept trial, designed to focus directly on reducing these two sources of delay, mindful that any improvements in help-seeking delays alone, for example, by increasing community awareness of psychosis, would be compromised if young people continued to be referred into a mental health service with prolonged delays in treatment response [17].

The intervention comprised two components, each designed to target a distinct part of the care pathway. The first was the introduction of a youth mental health care pathway, providing rapid engagement and assessment for young people 16–25 years and seamless transfer to EIS without need for further assessment by secondary MHSs [18, 19]. The purpose of this new youth team was to ensure all first episode cases of psychosis identified were given direct access to EIS, with sensitive management in a youth friendly context to reduce disengagement. The second component of the intervention, focused on improving help-seeking behaviour, was implementation of a public health campaign, to run alongside the new youth mental health team. Using our website www.youthspace.me as the central hub of the campaign and the strapline *‘Don’t turn your back on the symptoms of psychosis’* the campaign aimed to raise awareness of psychosis in the local community, improve knowledge of early warning signs and provide information to families and young people about when, where and how to seek help.

### Hypothesis

The principal hypothesis to be tested was whether introduction of a new youth access pathway for first-episode psychosis, enabling direct access to EIS and removing the need for interim contact with secondary MHSs, running alongside a psychosis awareness public heath campaign, would significantly reduce DUP in Birmingham, UK.

## Methods

### Design

This was a quasi-experimental, proof-of-principle prospective study comparing a specified area in south Birmingham (the intervention area), whereby a new youth access service (YouthSpace) was about to be introduced, with a comparable control area in north Birmingham, UK, providing detection as usual.

Incident cases of first-episode psychosis from both areas were identified and their DUP and care pathways measured over the duration of the trial (July 2011 – Dec 2013). We also used extensive recent DUP data from the NIHR National EDEN study (**E**valuating & **D**eveloping **E**arly Interventio**N** Services) which was available to define the baseline [20].

### Sample

Birmingham is the second most populous city in Britain with a high degree of cultural and religious diversity and ranked the third most deprived city in England [21, 22]. A breakdown of the population profile of gender, age range and ethnicity for both the intervention and control areas is shown in Table 1. The control and intervention areas whilst in close proximity to one another did not share EIS personnel.

**Table 1:** Population profile of Intervention and Control Areas

	Intervention area (10 Wards)	Control area (9 Wards)
Population	249,813	217,500
Gender	120,731 (48.3 %) Male	105,147 (48.3 %) Male
	129,082 (51.6 %) Female	112,353 (51.6 %) Female
Persons aged 0–24	91,641 (36.6 %)	68,210 (31.3 %)
Ethnicity	193,612 (77.5 %) White British	175,946 (80.8 %) White British
	26,715 (10.6 %) Asian/Asian British	18,344 (8.4 %) Asian/Asian British
	14,073 (5.6 %) Black/Black British	13,570 (6.2 %) Black/Black British
	11,873 (4.7 %) Multiple Ethnicity	8,340 (3.8 %) Multiple Ethnicity
	3,540 (1.4 %) Arab/Other	1,300 (0.5 %) Arab/Other

### Measures

DUP is routinely collected for all clients with a first episode of psychosis at entry into EIS. It is calculated using a combination of retrospective assessment of positive and negative symptoms of psychosis, client interview and electronic care records. This is based on the method described by Larsen et al. [23] and used in our research [16].

### Structured Clinical Interview for Positive and Negative Syndrome Scale (SCI-PANSS)

This is a regularly used clinical assessment of psychosis [21], comprising 30 items rating severity of positive symptoms (7 items; range 7–49), negative symptoms (7 items; 7–49) and general psychopathology (16 items; range 16–112) (Additional file 1). It takes approximately 30–45 min to complete. It has good reliability, criteria-related validity and construct validity [23].

### DUP and component delays

We defined **DUP** as the time period between *onset of psychosis* and the *onset of criteria treatment*. The definition of these time points as used in our previous research [16] is as follows:

- *Onset of psychosis:*
  (i) One positive symptom (SCI-PANSS positive 1 to positive 7) rated as moderate or above (4 or above):
    or:
  (ii) A cluster of positive symptoms (positive 1 to positive 7) reaching a total rating of 7 or more (not rating absent symptoms)*.
    *The cluster required at least one of the symptoms positive 1, positive 2 or positive 3 to qualify as onset of psychosis.
- *Onset of criteria treatment (OCT):* the date when adequate treatment commenced (as recorded in healthcare records), which was:
  (i) Adhering to dosage levels recommended by British National Formulary [24];
    and either:
  (ii) Continued adherence for a period of at least 1 month, or
  (iii) Leading to significant reduction in symptoms as measured by SCI- PANSS [25]. (This option, however, was, in practice, never used.)

#### Delay in help-seeking

Defined as the interval between the *onset of psychosis and first help-seeking contact.* Where individuals were already in contact with services (for example for prodromal symptoms) at the onset of psychosis, signifying help-seeking had already occurred, delay in help-seeking for psychosis was set to 0.

#### Delay within MHSs

The interval between the *first contact with secondary MHSs* after the onset of psychosis and the *onset of criteria treatment* (OCT). Where the individual was already in contact with services (for example for symptoms presented during the prodrome), the contact, which coincided with the time of onset of psychosis, was taken as the onset of MHSs delay.

#### Delay in accessing EIS

The interval between the *first help-seeking contact* and *acceptance by E*IS. The standard method of calculating DUP is not affected by any delay in accessing EIS and is calculated independently of DUP.

### Pathways to care interview

This interview follows the method of Gater et al. [26]. Systematic information about an individual’s care pathway is gathered from a combination of direct interview and electronic care records, regarding source, sequence and timing of help-seeking by clients and their families. This includes help-seeking contacts, the main problems presented and treatments offered.

Following this interview, data were then synthesised onto visual ‘route timelines’; sequencing help-seeking contacts, referrals made, diagnoses offered, treatment provided and outcomes.

All interviews were conducted by trained graduate psychologists embedded in each EIS team. Six-monthly checks on their assessment reliability, consisting of submission of five timelines and DUP calculations to DUP co-ordinators in Birmingham, for concordance and standardisation of calculation were conducted; Kappa or intra-class *r* >0.75 required. This followed the methodology used in the multi-site National EDEN study [20].

Each interview took approximately 1 h to complete.

## The intervention

**Fig. 1:**
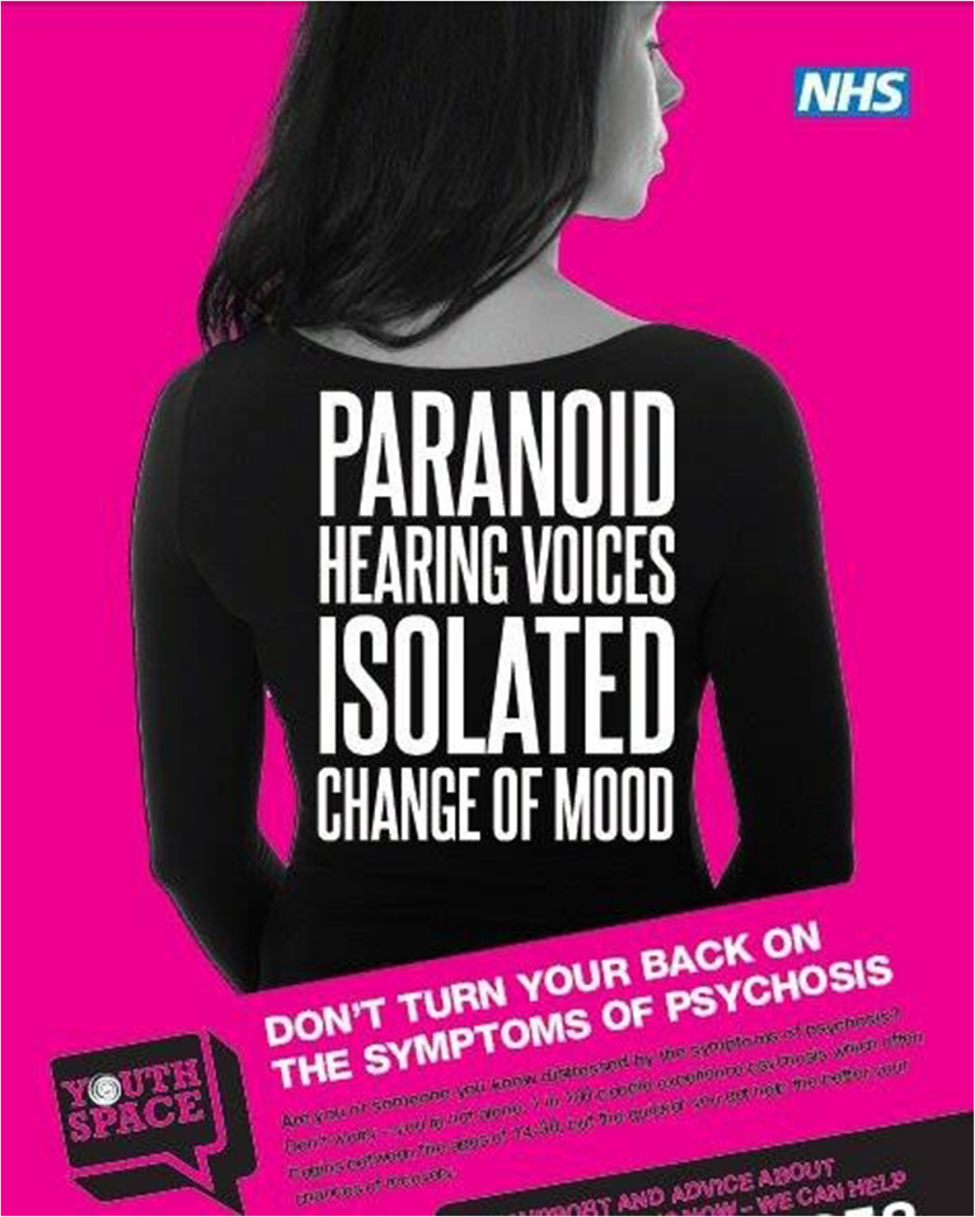
Example of campaign poster
      YouthSpace advertisements were placed in monthly, bi-monthly and quarterly newspapers and magazines delivered free to homes and appeared on 6 community websites, 10 library web-pages and in local GP surgeries.
      YouthSpace leaflets and postcards were distributed on high streets and in shopping centres and mail shots of these leaflets delivered to individual homes in ‘difficult-to-reach’ areas with no formal community hub.
      A variety of community, educational and NHS events were attended by research staff, clinicians and youth advisors.
    ***Psychosis Information Line***
      A telephone information line was also included on all campaign material. This provided families and young people with an alternative method of seeking help and information about psychosis. This daily service was staffed by trained researchers who followed a clear protocol developed in collaboration with the YouthSpace clinical team, regarding referral procedures and governance issues.
    ***Youth Advisors***
      Our youth advisors (‘Youthboard’), are young people with experience of MHSs. Throughout the campaign they provided great insight and expertise in designing and developing the YouthSpace website and campaign resources including photography, films and blogs. They also attended several community events with the research team.
    ***Psychosis Awareness Training***
      Bespoke training events designed specifically for the individual needs of emergency services, youth, community groups and employment and education agencies were delivered by the campaign team with the aim of outreaching into the community, improving knowledge and awareness of early warning signs of psychosis and creating a broad network of organisations and individuals with which to increase the scope of the campaign.

1. **Youth mental health care-pathway**
  The youth access pathway into MHSs (‘YouthSpace’) was launched in July 2011. Following promotion of YouthSpace by clinical staff to all GP surgeries in the intervention area, the new service was embedded within two of the Trust’s largest Community mental health teams (CMHT’s) located in a specified area in the south of the city. The operational principles of YouthSpace provided direct access to EIS for those presenting with symptoms of psychosis, offering: prompt clinical assessment in a youth appropriate setting; rapid access and expert assessment based on formulation principles; home visits in cases of repeat non-attendance (‘Did not attend’: DNA); provision of a brief CBT based intervention, where appropriate, as a default; situating the GP as ‘default prescriber’, with expert support from a consultant psychiatrist; implementation of clearly defined interface roles between the clinical service and primary care (‘collaborative care’); and prompt response in cases of crisis via established channels [18, 19].
2. **Mental health care pathway monitoring**
  To provide a snapshot of the typical numbers of cases presenting with possible first-episode psychosis symptoms referred to the Birmingham mental health service (Birmingham & Solihull Mental Health Trust) and their subsequent care pathway, live monitoring was conducted throughout the intervention period, using the Trust electronic case recording systems.
3. **Community psychosis awareness campaign**
  Six months following the introduction of YouthSpace, our public health campaign was launched in the intervention area, with the aim of improving community knowledge and awareness of first-episode psychosis and reducing help-seeking delays. The development and implementation of the campaign followed the ‘Precede-Proceed’ public health model framework [27] and included on-going assessments of context and setting to ensure a responsive, stratified ‘knowledge-transfer’ approach. Initial findings from the ‘precede’ phase of our programme enabled comprehensive assessment, planning, piloting and target-setting of the campaign and included both patient and public involvement, with regular consultations with an advisory board of young people (the ‘YouthBoard’), users of the mental health services and their families.
  The framework was further underpinned by two theoretical models which addressed the cognitive and contextual determinants of health behaviour change, the Trans-theoretical/Stages of Change model [28] and the MINDSPACE framework [29], the latter arising from behavioural economics and widely employed by UK policymakers [30].
  Our previous research into DUP in Birmingham [16] included qualitative interviews with young people referred to EIS who had experienced long DUP (>6 months) and their carer’s. These interviews highlighted the key roles that parents and family networks play in initiating help-seeking for psychosis and directly informed the rationale of our campaign, ensuring a ‘family-focused’ approach. The campaign comprised the following components:
  This methodology was vital in clarifying the process of behaviour change with regard to improving help-seeking behaviour and community response to public health initiatives. It also highlighted the key roles that parents/carers play in initiating the help-seeking process culminating directly in the development of a ‘family-focused’ campaign, comprising of the following components:
    ***Publicity & community engagement***
      All promotional material used for publicity included a link to our website, www.youthspace.me which served as the central information hub for the campaign.
      YouthSpace posters (example: Fig. 1) were displayed in high-use community settings including local bus services and shopping centres, supermarkets, employment offices, community and youth groups, leisure centres, coffee shops and fast-food outlets.

## Statistical analysis

### Principal analysis

All incident cases of first-episode psychosis from the intervention and control areas were identified via the relevant Birmingham EIS. All cases accepted into these teams served as the sampling framework. DUP and care pathways were assessed throughout the duration of the trial. Data from the National EDEN study in Birmingham, recorded over two years (*n* = 178), was used for baseline comparison of DUP.

The principal analysis was based upon a mixed model (ANOVA), including *time period* (pre- intervention period and intervention period), *area* (North (control) or South (intervention) and the *interaction between period and area* (to estimate the intervention effect). The response variable (DUP) was log_e_ transformed, and thus the results describe a ratio or relative reduction of DUP by area. Statistical analyses were conducted in Proc Glimmix, SAS 9.4 (SAS Institute, Cary NC).

### Secondary analyses

#### Mental health care pathway monitoring

A snapshot of young people experiencing first-episode psychosis referred into our MHS was monitored throughout the trial to provide us with a picture of the typical care pathways experienced by other young people presenting with first-episode psychosis.

#### Website hits, information line calls and campaign activity

The number of website visits to www.youthspace.me and calls to the information line and campaign activities were also monitored throughout the trial.

## Results

As expected, our data was skewed due to the existence of a number of outliers with very long DUPs. This gave rise to large standard deviations and a discrepancy between means and medians. In light of this we have reported median results. Mean, median and standard deviations for baseline data from national EDEN study (19) are provided in Tables 2 and 3. Data for the intervention and control areas are provided in Tables 4 and 5.

**Table 2:** Historical demographics for EIS clients in intervention and control areas (National EDEN)

	Control area (*n* = 98)	Pre-intervention area (*n* = 80)
Gender	29 (29.5 %) Female	18 (22.5 %) Female
	69 (70.4 %) Male	62 (77.5 %) Male
Mean age	22.2 years	22.5 years
Ethnicity	52 (53 %) White British	45 (56.2 %) White British
	21 (21.4 %) Asian Pakistani	13 (16.2 %) Asian Pakistani
	6 (6.1 %) Black (other)	5 (6.2 %) Black Caribbean
	3 (3 %) Asian Indian	4 (5 %) Black African
	3 (3 %) Black African	3 (3.7 %) Asian Indian
	3 (3 %) Black Caribbean	3 (3.7 %) Mixed heritage (White & Black Caribbean)
	3 (3 %) Mixed heritage (White & Black Caribbean)	2 (2.5 %) Mixed heritage (White & Black African)
	2 (2 %) Asian (other)	1 (1.2 %) Mixed heritage (White and Asian)
	1 (1 %) Mixed heritage (other)	1 (1.2 %) Black (other)
	1 (1 %) White Irish	1 (1.2 %) Asian Bangladeshi
	1 (1 %) Asian Bangladeshi	1 (1.2 %) White Irish
	1 (1 %) White (other)	1 (1.2 %) White(other)

**Table 3:** Baseline DUP data for EIS clients in pre-intervention and control areas (National EDEN)

		Help-seeking delay	Delay within MHSs	Delay in reaching EIS	DUP	*N* = 178
Control area	Mean	**41.07**	**109.40**	338.55	**213.27**	98
	Median	0	2.5	90	31.5	
	St Dev	132.81	389.46	583.65	459.03	
Pre-Intervention area	Mean	**95.40**	**118.36**	396.75	**284.54**	80
	Median	4.00	19.00	93.50	71.00	
	St Dev	238.43	272.01	772.87	482.13	

The bold text is to highlight the mean scores

**Table 4:** Demographic characteristics of EIS clients in intervention and control areas

	Control area (*n* = 74)	Intervention area (*n* = 77)
Gender	28 (38 %) Female	25 (32 %) Female
	46 (62 %) Male	52 (68 %) Male
Mean age	21.6 years	22.5 years
Ethnicity	29 (39 %) White British	37 (48 %) White British
	1 (1 %) White Irish	0 (0 %) White Irish
	1 (1 %) Asian Bangladeshi	3 (4 %) White – Other
	2 (3 %) Asian-Indian	2 (3 %) Asian Bangladeshi
	5 (7 %) Asian –Other	2 (3 %) Asian-Indian
	18 (24 %) Asian Pakistani	3 (4 %) Asian –Other
	2 (3 %) Black African	5 (6 %) Asian Pakistani
	2 (3 %) Black Caribbean	1 (1 %) Asian British Pakistani
	4 (5 %) Mixed White & Black Carribbean	4 (5 %) Black African
	3 (4 %) Other Ethnic Group	4 (5 %) Black Caribbean
	7 (9 %) Missing	6 (8 %) Mixed White & Black Carribbean
		2 (3 %) Mixed – Other
		1 (1 %) Mixed White Asian
		7 (9 %) Missing

**Table 5:** DUP for EIS clients in intervention and control areas during trial (July 2011 – Dec 2013)

		Help-seeking delay	Delay within MHSs	Delay in reaching EIS	DUP	*N* = 151
Control area	Mean	**116.97**	**124.19**	162.30	**216.43**	74
	Median	11.50	21.00	44.00	79.50	
	St Dev	229.02	216.45	242.84	335.86	
Intervention area	Mean	**41.49**	**42.32**	130.57	**103.82**	77
	Median	1.50	6.50	40.50	39.00	
	St Dev	105.93	86.74	225.89	155.00	

The bold text is to highlight the mean scores

### The samples

#### Baseline data

Demographic data from National EDEN study [19], for pre-intervention and control areas, is shown in Table 2. The majority of young people in these areas, at this time, were male, of White British or Asian Pakistani heritage, with a mean age of 22.5 years.

Clients from the control area had a median DUP of 32 days (mean 213 days), MHS delay of 3 days (mean 109 days), help-seeking delay of 0 days (mean 41 days) and a delay in reaching EIS of 90 days (mean 339 days) (Table 2).

Those from the pre-intervention area had a median DUP of 71 days (mean 285 days), MHS delay of 19 days (mean 118 days) help-seeking delay of 4 days (mean 95 days) and delay in reaching EIS of 94 days (mean 397 days).

A Mann–Whitney *U* Test revealed the difference between median DUP in National EDEN study, for control and pre-intervention areas, was significant (0.009).

#### Incident cases

The demographic profile of cases from the intervention and control areas is shown in Table 4. The majority of young people in these areas were male with a mean age of 22.5 years and of White British or Asian Pakistani heritage.

**Table 6:** Typical pathway to EIS in pre-intervention and control areas 12-months prior to trial (July 2010 – July 2011)

Pathway	Pre-intervention (*N* = 48)	Control (*N* = 109)
Crisis team	7 (15 %)	38 (35 %)
Community mental health team	15 (31 %)	42 (39 %)
CAMHS	9 (19 %)	0
Primary Care	4 (8 %)	0
A youth support team	2 (4 %)	5 (5 %)
Transfer from out-of-area EIS team	5 (10 %)	3 (3 %)
Early detection team	0	8 (7 %)
Hospital	4 (8 %)	0
Other	2 (4 %)	13 (12 %)

A total of 189 individuals entered EIS care in the control (*n* = 98) and intervention (*n* = 91) areas during the intervention period (July 2011 - December 2013). 24 cases were subsequently removed from the control group and 10 from the intervention group due to having received EIS treatment from a previous EIS team in another area. Four further cases were removed from the intervention group due to incomplete data which resulted in inability to calculate an accurate DUP. This left a final total of 74 individuals from the control area and 77 in the intervention area with fully complete DUP data.

In the control area, post-intervention median DUP was 80 days (mean 216 days), MHS delay of 21 days (mean 124 days), help-seeking delay of 12 days (mean 117 days) and delays in reaching EIS of 44 days (mean 162 days) (Table 5).

For those in the intervention area, post-intervention median DUP was 39 days (mean 104 days)), MHS delay of 7 days (mean 42 days)), help-seeking delay 2 days (mean 41 days) and delay in reaching EIS 41 days (mean 131 days)).

#### Principal analysis

Our statistician conducted a robust examination of the data and used log_e_ transformation of the response variable (DUP) as the optimal method to handle the skew in our data. This is a standard approach when analysing DUP data.

The relative reduction in DUP in the intervention area, accounting for baseline period as a random intercept term, was 0.735 (95 % CI 0.348 to 0.893; *p* = .0039) describing a clear relative reduction in DUP in the intervention area, having accounted for temporal change and baseline effects.

#### Care pathways to early intervention teams: intervention vs control areas

In the 12-months prior to the implementation of the intervention (July 2010 – July 2011: 12-months), a total of 109 EIS referrals were made in the control area. Thirty-nine percent of these referrals were made via CMHT or crisis team (35 %). A total of 48 EIS referrals were made in the intervention area during this time. The majority of these referrals came through CMHTs (31 %), Child & Adolescent Mental Health Teams (CAMHS) (19 %) or crisis teams (15 %) (Table 6).

During the intervention (July 2011 – Dec 2013: 30-months) a total of 74 EIS referrals were recorded in the control area. Typical pathways for referrals were through CMHTs (36 %) and crisis teams (32 %) (Table 7). In the intervention area a total of 77 referrals to EIS were recorded. Whilst 16 (21 %) of these referrals came through the typical pathway (CMHT), 17 (22 %) came directly through the new pathway YouthSpace team. These young people had a mean DUP of 149 days, HS delay of 42 days and MHS delay of 68 days (Table 8).

**Table 7:** Typical pathway to EIS in intervention and control areas during trial (July2011 – Dec 2013)

Pathway	Intervention area (*n* = 77)	Control area (*n* = 74)
Crisis team	28 (36 %)	24 (32 %)
Assertive Outreach Team	0	1 (1 %)
Casualty	0	1 (1 %)
Generic mental health team	16 (21 %)	27 (36 %)
A youth support team	3 (4 %)	6 (8 %)
YouthSpace	17 (22 %)	0
Child & Adolescent Mental Health Team	4 (5 %)	3 (4 %)
Primary Care	3 (4 %)	2 (3 %)
Psychiatric Hospital	3 (4 %)	7 (9 %)
Early detection team	0	1 (1 %)
Other	3 (4 %)	2 (3 %)

**Table 8:** DUP for those who referred to EIS via the YouthSpace pathway (*n* = 17)

	Help-seeking Delay	Delay within MHSs	Delay in reaching EIS	DUP	*N* = 17
Mean	**42.25**	**68.46**	125.68	**149.25**	
Median	0.5	34	31.5	91.5	
St Dev	109.56	84.74	199.43	161.58	

The bold text is to highlight the mean scores

### Secondary analysis

#### Mental healthcare pathway monitoring

Thirty two young people from the intervention area were referred into our MHS with clear psychotic symptoms during the intervention period. 16 (50 %) were subsequently referred to EIS during this time experiencing a median delay of 66 days (mean 126) in referral to EIS. 16 (50 %) of referrals, however, were not referred to EIS, instead remaining with generic mental health teams. At time of writing (March 2015), 11 (69 %) of these cases had subsequently been discharged from these teams due to ‘completed care’ [3], not attending appointments [3], not responding to communication [2], declining assessments [1], transfer [1] or deemed unsuitable [1]. At the time of writing, 5 (31 %) were continuing their care with a generic mental health team**.**

#### Website hits

There were a total of 24,813 website hits on during the intervention. Of these, 8,026 (32.3 %) were visits to the psychosis information page http://www.youthspace.me/search?q=psychosis.

#### Information line calls

Twenty eight calls were made to the information line during the intervention, an average of 1 call per month. Thirteen callers (46.4 %) had been made aware of the information line through local advertising. The vast majority (92.8 %) of callers to the information line, however, were either not from the intervention area, were already in contact with MHSs, were out of YouthSpace age range or did not meet EIS criteria for first-episode psychosis. These callers were signposted to the appropriate services for their particular needs.

Sixteen (57.1 %) calls were from females, 10 (35.7 %) from males and 2 (7.1 %) were unknown (callers failed to speak once the call had been answered). Twelve calls (42.8 %) were for self-help, 8 (28.5 %) were from carers requesting help for a family member, 6 calls (21.4 %) were from support/community workers and 2 calls (7.1 %) were unrelated to mental health issues.

Fifteen callers (53.5 %) rang to enquire about positive symptoms of psychosis, 8 (28.5 %) about depression. Only 2 of those enquiring about positive symptoms (13.3 %) met the criteria for referral to YouthSpace (age appropriate and experiencing psychotic symptoms).

#### Awareness campaign activity

Of the 95 campaign activities, more than half (52.6 %) were community events such as farmers markets, festivals and fun days. A third of activities were leaflet drops (33.6 %) with postcards and leaflets distributed in local shops, businesses and GP surgeries. Other activities included presence at 5 NHS related events, 3 bus advertising promotional events, 3 training events (with emergency services and youth organisations), 1 local radio appearance and a 1 mail shot (using Royal Mail) to a hard to reach area which had no central shopping area or high street**.**

## Discussion

Following our pragmatic, quasi-experimental trial targeting two specific components of the care pathway principally responsible for long DUP in this healthcare context, help-seeking delay and delay within MHSs [16], we observed a reduction in median DUP in the intervention area, from 71 days (mean 285) to 39 days (mean 103). The data from the control area was stable. The reduction in DUP was apparent in both of the component delays we targeted, in line with our hypothesis.

In the National EDEN (historical) data we observed a significantly longer DUP in the pre-intervention area, giving us, if anything, an even greater mountain to climb in our intervention area during the trial. However, by the end of the intervention period we saw a reversal of this situation, DUP in the intervention area was more than halved, suggesting that any historical differences were not responsible for the effect we observed.

Our study benefitted from the presence of long-standing early intervention services, which manage all incident cases in Birmingham and in our previous paper we showed that access to these was highly correlated with receipt of criterion treatment [16]. Whilst our findings did not focus on demonstrating that this reduction in DUP was associated with any improvement in psychotic symptoms, our findings may support the link between extended DUP and poorer treatment outcomes which has been validated in previous systematic reviews [1].

The question is raised as to whether the reduction in delay within the MHS arose in part or entirely due to the introduction of the YouthSpace service. During the intervention a total of 77 EIS referrals were made in the intervention area; almost one quarter of these referrals (22 %) through the new YouthSpace pathway. The median DUP for young people referred via our new service was 91 days, with a MHS delay of only 34 days.

Interestingly, these delays were fractionally longer than those observed in the intervention area overall. Further examination of the care pathways of the YouthSpace referral group revealed 2 of them with excessive MHS delays of almost 9-months and subsequent DUPs of around 11 months. Their MHS delays were due to engagement issues and being retained by CMHTs, despite psychotic symptoms. Removing these two cases MHS delay for the rest of the YouthSpace group revealed that, on average, their median MHS delay was 22 days (mean 38 days) with DUP of around 70 days (mean 70 days). This suggests that the introduction of our new pathway may have played an important part in the reduction in DUP.

### EIS Delay

Interestingly when compared to our historical National EDEN data, both intervention and control areas showed significant reductions in EIS delay (from median of 94 days (mean 397 days) to median 41 days (mean 131 days) in the trial area; from median of 90 days (mean 339 days) to median 44 days (mean 162 days) in the control area). This is, perhaps, a reflection of the growing awareness of EIS across MHSs in general in the last few years’. Referrer’s knowledge and acceptability of specialist services is vital for a referral to occur and this may have been an important factor in our findings.

It was interesting to note a greater number of referrals to EIS during the pre-intervention period in the control area compared with those received in the intervention area. This may suggest either, i) the control area was struggling with capacity issues, or, ii) there was a good infrastructure in place for referral to EIS. Given that help-seeking delays and delays to EIS in the control area were short, in comparison, this may suggest the latter is true. Delays in accessing EIS in the control area was further reduced following the intervention period, suggesting that these capacity issues may have become less of an issue during this time; a stark increase in help-seeking delay and a consistently long DUP, however, implies likely issues with engagement of young people and their families with services.

As well as examining care pathway delays and DUP in our intervention and control areas, we also engaged in a live monitoring exercise during the trial, examining all MHS referrals, with the aim of following the care pathways of young people, over a period of 6-months, who presented to our MHS with psychotic symptoms. Whilst half of cases went on to be referred to EIS within the 6-month timeframe, half of them were not and, instead, were cared for by generic mental health teams. Why these young people were not deemed suitable for referral to EIS is unclear, but the consequences of not doing so resulted in 69 % of them being discharged from the teams due to not attending appointments, failing to respond to communication and declining assessments.

### Psychosis awareness campaign

Although engaged in a wide variety of activities throughout the campaign, placing ourselves in high-activity community settings, the question arises as to whether such activities were directly responsible for the reduction in help-seeking delays we observed. Our presence in these sites, however, and the response of the communities we engaged with, revealed to us the importance of operating at grassroots level in the drive to improve awareness of symptoms and knowledge of help-seeking sources. We found that placing ourselves in normal family situations, for example, in the supermarket and on high streets, enabled frank discussions about psychosis and mental health to take place.

Despite the provision of our information line, it was infrequently used, with an average of only one call per month. With limited research staff the information line was, unfortunately, only available each afternoon and this may have had an impact on usage. A message service was made available for callers who rang out of hours, however, in hindsight this may not have been very helpful for those wanting to discuss sensitive issues and callers, unable to speak to someone in the first instance, may have been dissuaded from calling back.

### Limitations

One of the components of DUP is delay in help-seeking which was defined as the period between onset of psychosis and first help-seeking contact. We acknowledge, however, that there is a group of young people who, despite seeking help for their mental health, may conceal their psychotic symptoms or may not be ‘picked-up’ by secondary healthcare professionals, and, consequently, not be referred to EIS and experience long DUP [31]. Our decision to set the help-seeking delay for those already in contact with services at onset to 0, will have excluded the nature of other delays experienced by this group.

Our live monitoring exercise identified an extremely vulnerable group of young people who, despite presenting with FEP symptomatology, were never referred to EIS. The DUP of this group would have had an impact on the overall DUP we observed in our study. However, as DUP is only calculated on entry into EIS, we have limited data with which to explore this group further.

The limited duration of our intervention (30 months in total) meant that a fully robust evaluation was compromised (it may be argued that interventions need much longer implementation before any real impact is visible). However, our intervention was prospectively designed and utilised a pre-specified analysis plan which enabled us to evaluate the delivery process, community response and acceptability, as it proceeded. Nevertheless, it was not a randomised controlled trial, which would have eliminated any confounds and increased our statistical validity, and therefore we cannot assume that the effects noted here are directly associated with our intervention.

Birmingham has a population of approximately 1085,400, and is split into 40 administrative areas or ‘wards’. The control and intervention areas were in the south and north of the city, approximately 9 miles apart. However, whilst we endeavoured to restrict campaign promotion to the intervention area, we understand that complete contamination prevention was unlikely and we may have not been completely able to ensure that certain elements of the campaign (such as bus advertising) were not leaked into the control area at some point. Nevertheless, this would only have reduced the observed differences and not invalidated our results.

Our limited analytics regarding website usage revealed that it was well used throughout the campaign, with consistently high numbers of hits on the psychosis specific pages. We are conscious of the growing importance of digital media in public health campaigns particularly in relation to young people but are aware that more detailed analytics, including details of characteristics of the visitors to the website, would have provided us with greater information regarding use and impact of the website.

### DUP – the whole picture?

DUP is defined as the time between onset of psychosis and onset of anti-psychotic medication and our previous research revealed that, for some young people, medication is often only prescribed when they reach EIS [16]. For many, however, medication is received *prior to contact* with EIS. Our study found that this occurred for 82 % of those from the control area and 70 % from the intervention area. The standard method of calculating DUP for such young people will not take into consideration any delays experienced in accessing specialist treatment teams. In light of this, research exploring DUP should ensure delays in accessing EIS are also considered, after all, EIS provide young people with a wide range of benefits, as documented by the Department of Health in 2011 highlighting the influence EIS teams have in reducing the likelihood of relapse or detainment under the Mental Health Act.

## Conclusion

Long DUP has been consistently shown to predict poorer outcome for those with first-episode psychosis, the first 6-months of treatment delay believed to be a critical period, which, if extended, can impair treatment response and recovery [3–8]. Indeed, a dose response relationship between DUP and clinical symptoms has been suggested [2]. This proof-of-principle trial did not include follow-up assessment of clinical symptoms or treatment outcome, issues which, given the evidence base, should be future priorities in DUP research. Yet, the real world design of our proof-of-principle study *was* evidence based; firmly placed in the local context, with strong external validity, high quality collection of data from a baseline (pre-intervention) period and inclusion of a prospective control region, factors which will have served to increase the robustness of our evaluation. In light of this, we believe our findings to be promising and suggest that the methodology we have used, focusing directly on primary sources of delay which disrupt the care pathways of young people in Birmingham, and responding to them with delay specific solutions, may help reduce DUP. Our successful experimental intervention, focusing on the community and use of youth-friendly digital media, has provided a generalizable methodology that should be applicable to a variety of healthcare contexts with differing sources of delay in the care pathways of their clients. Longitudinal trials which include evaluation of clinical symptoms and treatment outcomes could now be implemented in order to evaluate further the extent of impact such interventions may have.

## Ethics and consent status

The trial was sponsored by Research and Innovation department at Birmingham & Solihull Mental Health Foundation Trust. Advice was sought from the National Research Ethics Service for the NHS who stated that our public health trial fell outside of the requirements for formal approval as: no consent or recruitment of service users was required; no identifiable data was to be specifically collected or used for evaluation; and the only data used would be anonymised DUP data and number of incident cases, both of which are routinely collected for all clients entering the specialist Early Intervention Service (for first episode psychosis) as part of their initial assessment.

## Availability of data and materials

The dataset and materials supporting the conclusions of this article are available in the CLAHRC-WM Research Team (University of Warwick) at Birmingham & Solihull Mental Health Foundation Trust (Research & Innovation Department) upon request from the authors.

## Additional file

Additional file 1: SCI-PANSS components. (DOCX 15 kb)

## Abbreviations

CAMHS: child & adolescent mental health services
CMHT: community mental health team
DUP: duration of untreated psychosis
EIS: Early Intervention Service
MHS: mental health services
National EDEN: **E**valuating & **D**eveloping **E**arly Interventio**N** Services
NIHR: National Institute of Health Research
