# Supplementary material for: Don’t turn your back on the symptoms of psychosis: the results of a proof-of-principle, quasi-experimental intervention to reduce duration of untreated psychosis
Source: BMC Psychiatry. 2016 May 4;16:127. doi: 10.1186/s12888-016-0816-7 (PMC4855493; doi:10.1186/s12888-016-0816-7)
Supplement: Additional file 1: — SCI-PANSS components. (DOCX 15 kb) [file 12888_2016_816_MOESM1_ESM.docx]

**File Name - Appendix 1**

**Title of Data – SCI-PANSS components**

**Description of Data – Details of Positive Symptoms, Negative Symptoms and General Psychopathology components for use when rating psychosis using the SCI-PANSS**

**SCI-PANSS components**

**Positive Symptoms**

- Delusions
- Conceptual Disorganisation
- Hallucinatory Behaviour
- Excitement
- Grandiosity
- Suspiciousness/Persecution
- Hostility

**Negative Symptoms**

- Blunted Affect
- Emotional Withdrawal
- Poor Rapport
- Passive/Apathetic Social Withdrawal
- Difficulties in Abstract Thinking
- Lack of Spontaneity & Flow of Conversation
- Stereotyped Thinking

**General Psychopathology**

- Somatic Concern
- Anxiety
- Guilt Feelings
- Tensions
- Mannerisms and Posturing
- Depression
- Motor Retardation
- Uncooperativeness
- Unusual Thought Content
- Disorientation
- Poor Attention
- Lack of Judgement and Insight
- Disturbance of Volition
- Poor Impulse Control
- Preoccupation
- Active Social Avoidance
